# Ferroptosis in vascular injury of critically ill patients: implications of gut microbiota regulation

**DOI:** 10.3389/fcimb.2026.1781905

**Published:** 2026-06-04

**Authors:** Huimin Hou, Jiyuan Gao, Mingda Wang, Mingjun Sun, Tuo Zhang, Lei Tian, Zeyu Yang, Man Chen, Guodong Lian, Wei Fang

**Affiliations:** 1 Department of Critical Care Medicine, Shandong Provincial Hospital Affiliated to Shandong First Medical University, Jinan, China; 2 Translational Medicine Research Center, Medical Innovation Research Division and the Fourth Medical Center of Chinese People’s Liberation Army (PLA) General Hospital, Beijing, China; 3 Department of Gastrointestinal Surgery, Shandong Provincial Hospital Affiliated to Shandong First Medical University, Jinan, China

**Keywords:** ferroptosis, gut microbiota, ICU, therapeutic strategies, vascular-related diseases

## Abstract

The treatment strategies for vascular injury-related diseases have made certain progress, but acute cardiovascular and cerebrovascular events, ischemia-reperfusion injury, and sepsis-induced vascular leakage still remain major causes of high mortality in critically ill patients. A deeper understanding of the pathogenesis of vascular-related diseases is crucial for developing more effective treatment methods. Ferroptosis, a recently discovered form of regulated cell death (RCD) distinct from apoptosis, necrosis, pyroptosis, and autophagy, has been shown to play a key role in the progression of vascular-related diseases. In particular, in sepsis-induced vascular leakage, inflammation and oxidative stress increase susceptibility to ferroptosis, leading to tissue damage and adverse outcomes. Inhibition of ferroptosis can significantly reduce the adverse outcomes caused by vascular injury, thereby improving the outcome of patients. This paper focuses on analyzing the molecular mechanisms through which ferroptosis influences vascular injury-related diseases, emphasizing its significance in ICU care. Additionally, the paper explores the role of gut microbiota dysbiosis in inducing ferroptosis, offering new insights into the gut-ferroptosis-vascular axis as a potential therapeutic target for critically ill patients. A deeper understanding of these mechanisms can pave the way for more personalized and effective treatments, ultimately improving patient outcomes and reducing mortality in this high-risk population.

## 1 Introduction

Vascular injury represents a substantial health risk, particularly in critically ill patients in the ICU, where it often leads to complex complications that significantly worsen prognosis (Gao et al., 2021; Königstein et al., 2021; Sabbatinelli et al., 2021; Schaubroeck et al., 2024). The pathophysiology of these conditions is multifactorial, involving inflammation, oxidative stress, dyslipidemia, and imbalances in the oxidative/antioxidant system (Cabrera de Bravo et al., 2005; Yang et al., 2021). In particular, in the context of sepsis, these mechanisms contribute to exacerbated vascular leakage, which further compounds vascular dysfunction and injury in ICU patients (Zhang et al., 2023). Although advances have been made in treating vascular injury-related conditions, acute cardiovascular events, ischemia-reperfusion injury, and sepsis-induced vascular leakage continue to be associated with high mortality rates, presenting a major public health challenge (Ho et al., 2022; Pohlan et al., 2022; Zhang et al., 2023). Therefore, a deeper understanding of the mechanisms driving vascular-related diseases is essential to develop more effective therapeutic strategies.

Ferroptosis, a form of regulated cell death driven by iron-dependent lipid peroxidation, has attracted significant attention due to its involvement in inflammation, oxidative stress, and lipid metabolism (Mao et al., 2020; Wiernicki et al., 2020; Tang et al., 2021). Ferroptosis has been implicated in the pathogenesis of various vascular-related diseases, including (AS), stroke, and Ischemia/Reperfusion Injury (Cui et al., 2021; Fang et al., 2023; Xiang et al., 2024). Emerging evidence also suggests that ferroptosis plays a pivotal role in ICU patients, where it may exacerbate vascular injury and influence the prognosis of critically ill individuals (Ling et al., 2023; Zhang et al., 2023). Ferroptosis in sepsis-induced (She et al., 2021) contributes to vascular damage by disrupting the endothelial barrier, increasing vascular permeability, and promoting tissue edema. Notably, the intestinal microbiota has been identified as a potential modulator of ferroptosis, affecting vascular injury through its regulation of gut health and metabolic processes (Deng et al., 2021; Kim et al., 2024). The interplay between the gut, ferroptosis, and vascular injury in ICU patients opens new avenues for targeted therapeutic interventions.

This review aims to explore the role of ferroptosis in vascular diseases, with a particular emphasis on ICU management. It will examine how ferroptosis contributes to vascular injury in critically ill patients, particularly in the context of sepsis and vascular leakage, and discuss the regulatory effects of intestinal microbiota on ferroptosis. Furthermore, potential treatment strategies, such as inhibiting ferroptosis and modulating intestinal microbiota, will be highlighted as promising therapeutic approaches for improving outcomes in vascular-related diseases and ICU patient care.

## 2 Mechanisms of ferroptosis-induced vascular injury

In 2012, Dixon et al. discovered that erastin, an inhibitor of the cystine/glutamate antiporter, could trigger a unique form of regulated cell death (RCD) that differs from apoptosis, necrosis, pyroptosis, autophagy, and other well-known types of cell death. This distinct form of RCD was subsequently named ferroptosis (Dixon et al., 2012). The molecular mechanisms and signaling pathways underlying ferroptosis are characterized by three key features: disruption of the System Xc-, alterations in lipid metabolism, and iron accumulation (Wang et al., 2020; Liang et al., 2022; Anandhan et al., 2023; Pope and Dixon, 2023). These hallmark characteristics drive both the initiation and progression of ferroptosis.

**Figure 1:**
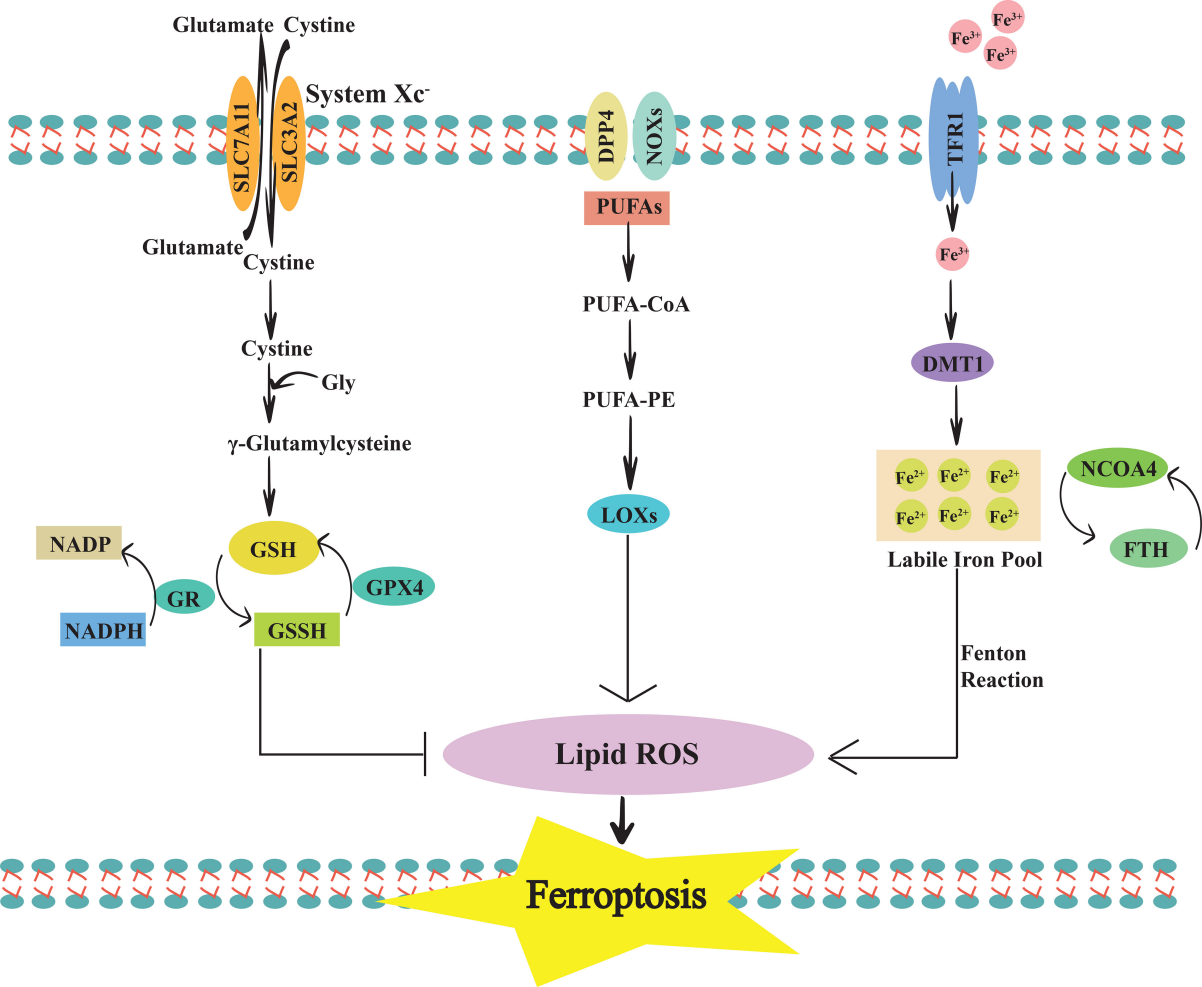
The molecular machinery and main signaling pathways of ferroptosis. Ferroptosis is characterized by several interconnected mechanisms, including the disruption of System Xc-, which impairs glutathione synthesis and leads to oxidative stress and ROS accumulation. Lipid metabolism dysregulation, particularly the peroxidation of polyunsaturated fatty acids (PUFAs) in cell membranes, further exacerbates oxidative stress. Iron accumulation plays a central role by catalyzing the Fenton reaction to generate ROS, damaging cell structures and promoting lipid peroxidation. Together, these factors drive ferroptosis through oxidative stress, lipid peroxidation, and iron metabolism imbalance, contributing to vascular dysfunction.

The impairment of System Xc- leads to an imbalance in cystine and glutamate transport (Li et al., 2022). This imbalance promotes the generation of reactive oxygen species (ROS), which in turn drives lipid peroxidation (Chen et al., 2021b). The resulting lipid peroxidation damages cellular membranes and exacerbates oxidative stress, which further accelerates the ferroptosis process (von Krusenstiern et al., 2023). At the same time, iron accumulation plays a central role by catalyzing the Fenton reaction, which generates additional ROS (Liang et al., 2022). This further induces cellular damage and propels ferroptosis. Moreover, the NADPH oxidase system, particularly NADPH oxidase 4 (NOX4), contributes significantly to ROS production, especially in the context of vascular injury (Wang et al., 2021b). When oxidative stress is present, NOX4 is activated and works synergistically with iron-dependent pathways, amplifying ROS production and accelerating lipid peroxidation (Park et al., 2021). This creates a positive feedback loop that intensifies the ferroptotic process. Together, these interconnected processes highlight the complexity of ferroptosis, which depends on the regulation of iron homeostasis, lipid peroxidation, and oxidative stress, with the NADPH oxidase system playing a key role in amplifying the damage (**Figure 1**).

## 3 Effect of ferroptosis on vascular injury in critically ill patients

**Figure 2:**
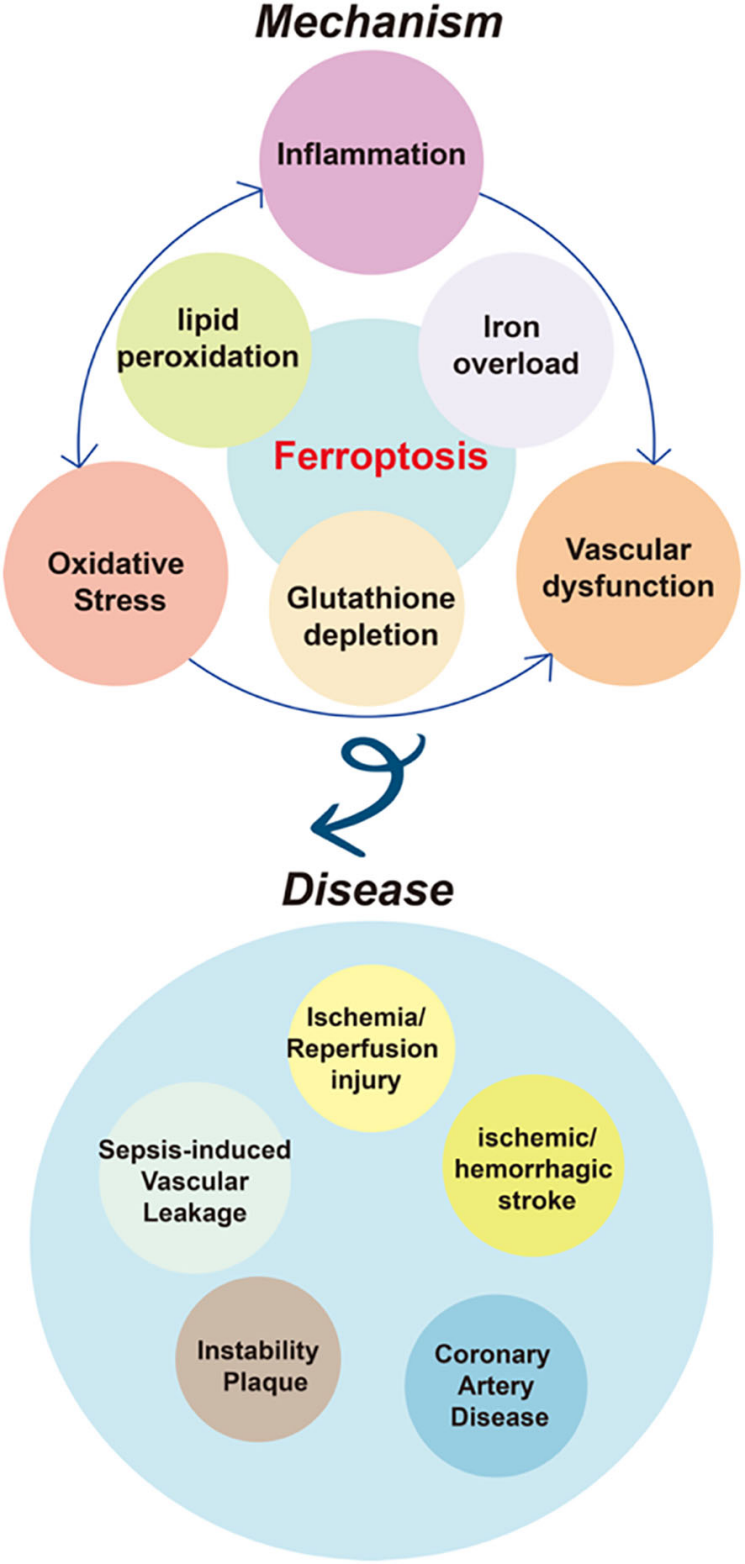
Ferroptosis is a key mechanism that induces vascular related diseases. Iron overload, lipid peroxidation, and glutathione depletion-induced ferroptosis lead to vascular dysfunction by triggering inflammation and oxidative stress, thereby contributing to the development of various vascular injury-related diseases, including ischemia-reperfusion injury, ischemic/hemorrhagic stroke, coronary artery disease, formation of unstable plaques, and vascular Leakage.

Ferroptosis has emerged as a critical factor in the pathogenesis of various diseases, particularly in critically ill patients with vascular damage (Stockwell et al., 2020; Chen et al., 2024). It plays a significant role in exacerbating tissue injury in conditions like ischemia/reperfusion injury, atherosclerosis-related cardiovascular and cerebrovascular events, and vascular leakage (Li et al., 2020; Wu et al., 2021; Tuo and Lei, 2024) (**Figure 2**). Understanding ferroptosis in these contexts is vital for developing targeted treatment strategies, particularly in the intensive care unit (ICU). This article will delve into the role of ferroptosis in these vascular diseases and its therapeutic potential.

### 3.1 Ischemia/reperfusion injury

Ischemia-reperfusion injury (IRI), also known as reoxygenation injury, can lead to severe damage to the blood supply, triggering a series of time-dependent clinical syndromes (Wu et al., 2018; Cai et al., 2022). IRI involves various pathological processes, including oxidative stress, inflammation, and intracellular iron accumulation, ultimately resulting in irreversible cell death through apoptosis and necrosis (Ding et al., 2023; Zhang et al., 2023). Recent research has highlighted ferroptosis, a novel form of regulated cell death, as a critical factor in the pathogenesis of IRI. Ferroptosis has emerged as an essential contributor to IRI in multiple organs, including the heart, brain, kidneys, and liver (Chen et al., 2021a; Lin et al., 2024; Liu et al., 2024).

Studies have shown that inhibiting ferroptosis can significantly reduce IRI. For instance, Ma et al. found that overexpression of SLC7A11 can reduce infarct size, improve pathological conditions, enhance cardiac function, and inhibit ferroptosis in a myocardial ischemia-reperfusion (MI/R) injury model (Ma et al., 2020). Similarly, Guan et al. demonstrated that activating the SLC7A11/GPX4 axis could inhibit ferroptosis in hippocampal neurons after IRI in gerbils (Guan et al., 2021). Furthermore, in a mouse model of intestinal ischemia/reperfusion-induced acute lung injury (IIR-ALI), ferroptosis inducers like erastin and liproxstatin-1(Lip-1) were shown to promote ferroptosis-related factors and contribute to IIR-ALI (Li et al., 2020). Additionally, Dong et al. identified that Nrf2 can inhibit ferroptosis by regulating SLC7A11 and HO-1 in IIR-ALI, which may represent a potential therapeutic strategy for IIR-ALI (Dong et al., 2020). Interestingly, the study also found that ferroptosis inhibitors, ferrostatin-1 and liproxstatin-1, can prevent ischemia-reperfusion injury in a middle cerebral artery occlusion (MCAO) model via reducing infarct volumes (Tuo et al., 2017). Together, these findings suggest that ferroptosis plays a crucial role in the pathophysiology of IRI. The role of ferroptosis in ischemia-reperfusion injury (IRI) provides us with new insights into the disease and potential intervention targets. By early identification of ferroptosis, particularly with timely intervention in ICU care, it is possible to effectively reduce the cellular and organ damage caused by IRI, decrease the risk of severe illness, and improve patient prognosis.

### 3.2 Stroke and cerebral ischemia

Stroke is one of the leading causes of morbidity and mortality worldwide, with ischemic stroke accounting for approximately 87% of all strokes (Saini et al., 2021). The incidence of ischemic stroke is rising globally, and it remains a major health concern, particularly in developing countries (Kuriakose and Xiao, 2020). Despite advances in acute management, the prognosis for ischemic stroke remains poor, with many patients experiencing long-term disabilities (Tu and Wang, 2023). The main pathological processes that influence the outcome of ischemic stroke include neuronal death due to ischemia, disruption of the blood-brain barrier (BBB), and a secondary inflammatory response (Okada and Suzuki, 2020). In addition to these classical mechanisms, ferroptosis has emerged as a key factor in the pathophysiology of hemorrhagic/ischemic stroke (Weiland et al., 2019). Research has shown that ferroptosis not only accelerates neuronal cell death but also exacerbates secondary inflammatory responses, which can further impede recovery and increase the risk of post-stroke complications, particularly in cases of ischemic brain injury involving large vessel occlusions or extensive ischemic regions (Fricker et al., 2018; Gao et al., 2024). Furthermore, in severe stroke patients, there is often a greater need for intensive care, including mechanical ventilation and the use of sedatives, which can complicate the regulation of oxidative stress and ferroptotic pathways (Dridi et al., 2020). The oxidative stress induced by these interventions, in combination with the already heightened risk of ferroptosis in the brain, makes targeting this pathway particularly relevant in critical care settings.

Despite this, recent studies have provided valuable insights into the role of ferroptosis in ischemic stroke. Chen et al. demonstrated that cerebral ischemia caused downregulation of ferritin, decreased SLC7A11 (an important transporter involved in iron homeostasis), and increased p53 expression in a middle cerebral artery occlusion (MCAO) mouse model of ischemic stroke. Overexpression of ferritin in this model inhibited ROS production, reversed the upregulation of p53, and improved cellular survival, highlighting the potential of targeting ferritin as a therapeutic strategy for ischemic stroke (Chen et al., 2021). Moreover, studies have also explored the role of ACSL4 (acyl-CoA synthetase long-chain family member 4), an enzyme involved in lipid metabolism and peroxidation. Overexpression of ACSL4 exacerbated ischemic brain injury in mice by promoting lipid peroxidation, neuronal death, and neuroinflammation. In contrast, the knockdown of ACSL4 reversed these effects, suggesting that modulation of ACSL4 expression may provide a promising therapeutic target for ischemic stroke (Cui et al., 2021). These findings underscore the critical role of ferroptosis in stroke-induced neuronal damage. Understanding and targeting ferroptosis in critically ill ischemic stroke patients could not only mitigate the immediate damage caused by stroke but also improve long-term recovery prospects by reducing brain injury, inflammation, and neuronal death, ultimately contributing to better functional outcomes.

### 3.3 Coronary artery disease and plaque instability

Coronary artery disease (CAD) remains one of the leading causes globally, with the majority of cases arising from coronary atherosclerosis (Joodi et al., 2020). The coronary arteries, which are essential for supplying oxygenated blood to the heart muscle, are particularly vulnerable to the development of atherosclerotic plaques. Key pathophysiological drivers such as inflammation, oxidative stress, and dyslipidemia play central roles in the progression of coronary atherosclerosis, significantly compromising vascular function (Kobayashi et al., 2018).

Notably, within the process of atherosclerosis, oxidative stress and inflammation act as critical catalysts for disease advancement. A key mechanism increasingly implicated in this setting is ferroptosis-an iron-dependent form of regulated cell death. Ferroptosis significantly contributes to endothelial dysfunction, which represents the initial step in atherosclerotic plaque formation (Wang et al., 2024). Specifically, iron accumulation promotes lipid peroxidation, damaging the endothelial lining and triggering the onset of plaque development (Xu et al., 2024). As lesions progress, vascular smooth muscle cells (VSMCs), crucial for maintaining structural stability of the arterial wall, also become susceptible to ferroptosis (Zhang et al., 2022). The loss of these cells leads to thinning and weakening of the fibrous cap, resulting in plaque destabilization and elevating the risk of rupture and thrombosis. Such events can precipitate acute clinical complications, including myocardial infarction (Ahmed et al., 2023; Stone et al., 2023). Furthermore, ferroptosis perpetuates a pro-inflammatory environment within atherosclerotic plaques. Iron-mediated lipid peroxidation activates potent inflammatory signaling pathways, establishing a vicious cycle of continuous cell death and inflammatory activation that accelerates plaque growth and enhances instability (Gao et al., 2024). This self-sustaining process underscores the role of ferroptosis as a critical amplifier of atherosclerosis and its clinical consequences.

For critically ill patients with advanced CAD, these processes underline the urgent need for timely and effective interventions to address the underlying pathophysiology. Early detection and targeted therapies aimed at modulating ferroptosis, inflammation, and oxidative stress could be crucial in preventing plaque rupture and improving outcomes for patients facing the most severe forms of this disease.

### 3.4 Sepsis-induced vascular leakage

Sepsis is a complex systemic response to infection that induces widespread inflammation, immune dysregulation, and oxidative stress. In this context, ferroptosis, an iron-dependent form of regulated cell death characterized by lipid peroxidation, plays a critical role in vascular injury and dysfunction (Shen et al., 2023). Endothelial cells, which line blood vessels, are particularly vulnerable due to their high content of polyunsaturated fatty acids in membrane phospholipids. Additionally, in septic conditions, excessive free iron is often released as part of the inflammatory response, promoting the Fenton reaction and amplifying oxidative stress (Liu et al., 2021). This oxidative stress overwhelms the cell’s antioxidant defenses, such as glutathione peroxidase 4 (GPX4), which normally prevents lipid peroxidation (Liu et al., 2023). When GPX4 activity is inhibited or exceeded, lipid peroxidation progresses, leading to endothelial cell death via ferroptosis (Qin et al., 2021; Li et al., 2025). Endothelial cells are crucial for maintaining the barrier function of blood vessels, and ferroptosis-induced damage to the endothelial monolayer increases vascular permeability, a hallmark of sepsis (Li et al., 2025). The loss of endothelial barrier integrity facilitates the extravasation of plasma proteins and immune cells, contributing to tissue edema and hypotension two defining features of septic shock (Tang et al., 2023). Furthermore, the disruption of vascular integrity exacerbates coagulation dysfunction, increasing the risk of disseminated intravascular coagulation (DIC) in septic patients (Luo et al., 2020).

Ferroptosis not only contributes to endothelial injury but also amplifies the inflammatory response in sepsis, creating a vicious cycle of vascular dysfunction and systemic inflammation (Xl et al., 2022). The release of damage-associated molecular patterns (DAMPs) and pro-inflammatory cytokines, such as TNF-α, IL-1β, and IL-6, from dying endothelial and immune cells further exacerbates systemic inflammation (Xiao et al., 2023; Hou et al., 2024). These inflammatory mediators increase the production of reactive oxygen species (ROS), which subsequently amplify oxidative stress and trigger ferroptosis in surrounding cells. This cycle of inflammation exacerbates vascular damage throughout the body, impairing the delivery of oxygen and essential nutrients to vital organs (Mallat et al., 2022). In critically septic conditions, where tissue perfusion is already compromised, the exacerbation of microcirculatory failure due to ferroptosis-induced vascular injury can worsen multisystem organ failure (MOF), a leading cause of death in sepsis (Li et al., 2023). Organs such as the lungs, kidneys, liver, and heart are particularly vulnerable to ischemic injury and reperfusion damage during sepsis. Moreover, emerging evidence suggests that ferroptosis in endothelial cells may trigger endothelial-to-mesenchymal transition (EndMT) (Ren et al., 2023). During this process, endothelial cells lose their endothelial characteristics and acquire a mesenchymal phenotype, contributing to vascular fibrosis and tissue remodeling. This phenomenon further impairs vascular function, promotes inflammation, and may lead to long-term sequelae in critically ill patients.

In summary, ferroptosis plays a pivotal role in the pathophysiology of sepsis by contributing to endothelial injury, vascular dysfunction, and the amplification of systemic inflammation. This vicious cycle of vascular instability, oxidative stress, and inflammation exacerbates organ damage, accelerates microcirculatory failure, and worsens outcomes in critically ill septic patients.

## 4 Role of gut microbiota in ferroptosis in critically ill patients

The gut microbiota, a complex community of microorganisms residing in the gastrointestinal tract, plays a crucial role in regulating numerous physiological processes, including metabolism, immunity, and oxidative stress (Zhang et al., 2024). Emerging evidence suggests that the gut microbiota may also play a pivotal role in modulating ferroptosis, a link that is particularly relevant in critical illness. The intricate crosstalk between the microbiome and ferroptosis occurs through multiple pathways, including regulation of host iron homeostasis, mitochondrial metabolism, and lipid peroxidation (Ding et al., 2025). A healthy gut microbiome contributes to the maintenance of iron homeostasis, which is essential for normal cellular function. However, under conditions of dysbiosis, this regulatory capacity is impaired, leading to the accumulation of free iron in tissues (Gu et al., 2024). This excess iron can catalyze the Fenton reaction, generating reactive oxygen species (ROS), which initiate oxidative stress and lipid peroxidation central mechanisms in the execution of ferroptosis (Lee et al., 2018).

A key methodological difference must be noted here, most existing studies linking gut microbiota to ferroptosis are based on murine models that can be used to establish causality by experimental interventions such as antibiotic depletion, sterile colonization, and metabolite supplementation. In contrast, human studies to date have been predominantly observational and can only demonstrate correlations. Although mouse models have provided mechanistic insights, direct causal evidence for the “gut microbiota-ferroptosis-vascular axis” in humans remains to be established.

However, emerging human observational studies are beginning to translate these preclinical findings. In a prospective study of 170 ICU patients with sepsis, serum ACSL4 (AUC = 0.7127) was associated with predicting sepsis mortality, and GPX4 levels were positively correlated with SOFA (Rho = 0.204, p*-*value = 0.027) and APACHE II (Rho = 0.233, p*-*value = 0.011) scores (Zeng et al., 2025). These data represent the first clinical evidence linking ferroptosis related proteins to sepsis diagnosis and prognosis in humans.

Beyond iron dysregulation, gut dysbiosis alters the production of key microbial metabolites that directly modulate ferroptotic pathways. Poor dietary habits and microbial imbalance promote the overgrowth of pathogenic gut microbiota, which synthesizes deleterious metabolites including lipopolysaccharide (LPS) and trimethylamine N-oxide (TMAO) (Du et al., 2025). LPS triggers TLR4/NF-κB/MAPK signaling cascades, fueling pro-inflammatory cytokine production that suppresses antioxidant defenses, particularly glutathione peroxidase 4 (GPX4) (Wei et al., 2020; Zhang et al., 2024). TMAO upregulates the endoplasmic reticulum stress PERK/eIF2α/ATF4 signaling pathway, thereby increasing reactive oxygen species (ROS) accumulation, DNA damage, and mitochondrial dysfunction (Zhang et al., 2025). Together, these metabolites synergistically induce ferroptosis in various cell types.

Concurrently, the dysbiotic microbiota reduces the production of beneficial metabolites such as short-chain fatty acids (SCFAs), tryptophan derivatives, and specific bile acids (BAs), which normally protect against ferroptosis (Neag et al., 2025; Wang et al., 2025). SCFAs, particularly butyrate, play a crucial role in maintaining gut barrier integrity and suppressing inflammation through nuclear factor erythroid-related Factor 2 (Nrf2)/glutathione peroxidase 4 (GPX4) or hypoxia-Inducible Factor 1-alpha (HIF-1α)/GPX4 pathways activation (Kelly et al., 2015; Chen et al., 2024). The loss of butyrate-producing genera such as *Faecalibacterium* and *Subdoligranulum* in critically ill patients compromises these protective mechanisms (Long et al., 2023; Cho et al., 2024). Bile acids, which are modified by the gut microbiota, act as signaling molecules by activating nuclear receptors, including the farnesoid X receptor (FXR), thereby influencing lipid metabolism and inflammatory responses (Chiang and Ferrell, 2022; Neag et al., 2025). Disruption of BA metabolism during dysbiosis may alter the regulation of ferroptosis-related genes. One of them is acyl-CoA synthetase long-chain family member 4 (ACSL4), a key enzyme that facilitates incorporation of polyunsaturated fatty acids (PUFAs) into membrane phospholipids and promotes ferroptosis (Zhang et al., 2025).

**Figure 3:**
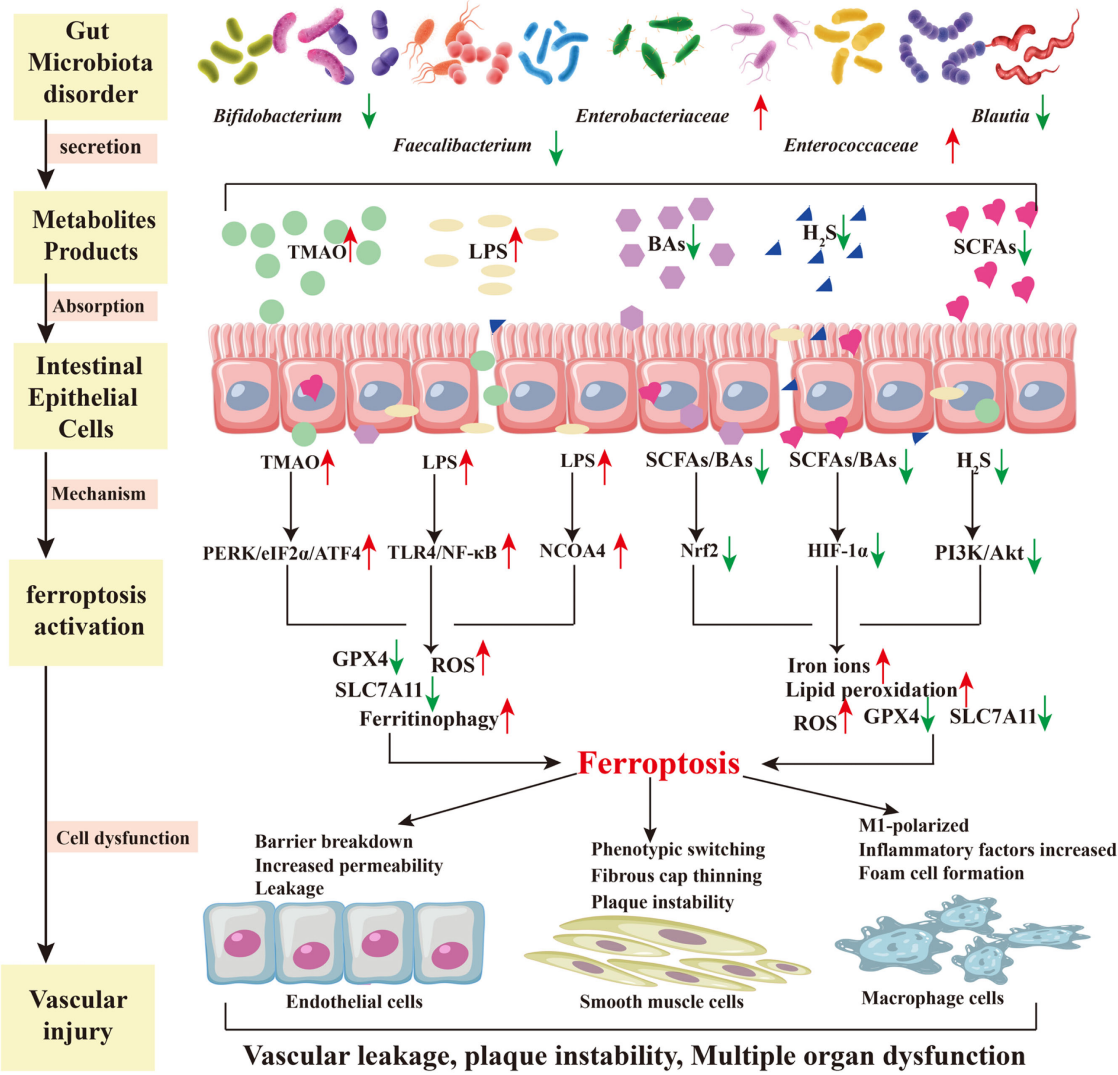
Gut microbiota dysbiosis regulates ferroptosis in vascular injury. Gut microbiota dysbiosis in critically ill patients alters the production of microbial metabolites. Pro-ferroptotic metabolites (TMAO, LPS) are increased, while anti-ferroptotic metabolites (SCFAs, BAs, H_2_S) are decreased. These metabolites enter the bloodstream and regulate ferroptosis-related signaling pathways in vascular cells: TMAO activates PERK/eIF2α/ATF4 pathway; LPS triggers TLR4/NF-κB or NCOA4 signaling; butyrate activates Nrf2/GPX4 axis or HIF-1α signaling; and H_2_S exerts antioxidant effects. Dysregulation of these pathways leads to ferroptosis in endothelial cells (barrier disruption, vascular leakage), vascular smooth muscle cells (plaque instability), and macrophages (inflammation), ultimately contributing to multiple organ dysfunction in critically ill patients. TMAO, trimethylamine-N-oxide; LPS, lipopolysaccharide; SCFAs, short-chain fatty acids; BAs, bile acids; H_2_S, hydrogen sulfide; PERK, protein kinase R-like endoplasmic reticulum kinase; eIF2α, eukaryotic initiation factor 2α; ATF4, activating transcription factor 4; TLR4, Toll-like receptor 4; NF-κB, nuclear factor kappa-B; Nrf2, nuclear factor erythroid-related Factor 2; HIF-1α, hypoxia-Inducible Factor 1-alpha.

Critically ill patients are highly susceptible to dysbiosis of the gut microbiota due to factors such as antibiotic use, physiological stress, altered nutrition, and prolonged hospitalization (Abenavoli et al., 2023). This dysbiosis is characterized by a shift in microbial composition, typically involving reduced alpha diversity, depletion of commensal taxa (e.g., Bifidobacterium, Blautia, Faecalibacterium), and expansion of potential pathogens including *Enterobacteriaceae* and *Enterococcaceae (*Victoria et al., 2022; Cho et al., 2024). Such alterations can exacerbate systemic inflammation, oxidative stress, and iron dysregulation, all of which are intimately associated with the induction of ferroptosis (**Figure 3**). The conceptual framework of a “gut microbiota-ferroptosis axis” has recently been proposed in various disease contexts, suggesting that abnormal iron metabolism and gut microbiota dysbiosis can promote each other, establishing a potential self-amplifying loop that drives tissue injury (Ding et al., 2025; Wang et al., 2025). Nevertheless, the specific causal relationships and molecular mechanisms linking gut microbiota dysbiosis to ferroptosis in critically ill patients remain an active and compelling area of ongoing research. Future investigations should prioritize dissecting the precise molecular crosstalk within this axis to identify novel therapeutic targets for improving outcomes in this vulnerable population.

## 5 Therapeutic strategies for ferroptosis and challenges

In light of the significant role ferroptosis plays in vascular diseases and other critical conditions, exploring potential therapeutic strategies to mitigate its harmful effects is essential. Two promising approaches are the use of ferroptosis inhibitors and the modulation of gut microbiota. Ferroptosis inhibitors, such as ferrostatin-1 and deferoxamine, aim to reduce iron accumulation, prevent lipid peroxidation, and stabilize cellular integrity, offering therapeutic potential in critically ill patients (Long et al., 2024). Additionally, targeting the gut microbiota through probiotics, prebiotics, and dietary interventions may help counteract ferroptosis by enhancing antioxidant defenses and reducing inflammation (Ma et al., 2025). These strategies, as discussed in the following sections, hold promise for improving outcomes in patients suffering from ferroptosis-related vascular injury and other complications.

### 5.1 Therapeutic strategies targeting ferroptosis inhibitors and their potential in critical illnesses

Any approach that inhibits ferroptosis works by targeting the specific mechanisms underlying this process, including iron chelation, inhibition of System Xc-, and blockade of lipid peroxidation pathways. Recent advances have identified major classes of preclinically established ferroptosis inhibitors, including iron chelators, radical trapping agents, lipophilic radical trapping antioxidants, and ninjurin-1 (NINJ1)-specific monoclonal antibodies (Maremonti et al., 2024). Several of these inhibitors have been evaluated in preclinical models of critical illness-related conditions (**Table 1**). However, their translational potential varies considerably due to differences in pharmacokinetic properties, target specificity, and safety profiles.

**Table 1:** Comparative analysis of major ferroptosis inhibitors: mechanisms, efficacy, and translational challenges.

Inhibitor class	Representative agents	Mechanism of action	Key advantages	Translational limitations	Safety in immunocompromised populations	Reference
Iron Chelator	Deferoxamine (DFO)	Binds Fe³^+^, inhibits Fenton reaction	FDA-approved; extensive clinical safety data	Short half-life (20–30 min); poor membrane permeability; requires continuous infusion	Generally safe, but caution in septic patients with anemia (chelator-induced iron deficiency may exacerbate anemia)	(Ihnat et al., 2000; Eckes, 2011; Liu et al., 2022)
	Deferasirox (DFX)	Binds Fe³^+^, suppresses ROS generation	Oral bioavailability; long half-life	Renal and GI toxicity; drug interactions in ICU	Renal and GI toxicity; higher risk in ICU patients with pre-existing renal dysfunction or on multiple medications	(Niño Taravilla et al., 2021)
	Deferiprone	Attenuates mitochondrial dysfunction, reduces ROS	Penetrates BBB	Risk of agranulocytosis; requires monitoring	Agranulocytosis risk – contraindicated in neutropenic ICU patients	(Cohen et al., 2000; Parakh et al., 2015)
Lipid Peroxidation Inhibitors	Ferrostatin-1 (Fer-1)	Traps lipid radicals; inhibits lipid peroxidation chain reaction	Potent; ferroptosis-specific	Poor water solubility; rapid degradation; no BBB penetration	No human safety data; theoretical risk: ferroptosis plays role in immune regulation; long-term inhibition may affect immune cell function	(Zilka et al., 2017; Shi et al., 2024)
	Liproxstatin-1 (Lip-1)	Inhibits mitochondrial lipid peroxidation; restores GPX4/GSH	Better metabolic stability than Fer-1	Poor solubility; limited BBB penetration	No human safety data	(Zilka et al., 2017; Cai et al., 2020)
Analogs	SRS 16-86	Stabilized Fer-1 analog; upregulates GPX4/xCT/GSH	Enhanced metabolic stability	Preclinical only; human safety unknown	No human data; preclinical models only	(Linkermann et al., 2014; Zhang et al., 2019)
	Ferfluor-1	Bioisosteric Fer-1 analog with real-time monitoring	Half-life extended 8—40 fold; penetrates BBB	Newly developed; no clinical data	Long-term safety demonstrated in animal studies at 50 mg/kg, but no human data	(Yan et al., 2025)
GPX4 Enhancers	N-acetylcysteine (NAC)	Glutathione precursor; induces GPX4 expression	Clinically available; multiple indications	Non-specific mechanism; benefits not attributable to ferroptosis alone	Generally safe; widely used in ICU for acetaminophen overdose, contrast nephropathy; rare hypersensitivity reactions	(Gupta et al., 2020; Zheng et al., 2025)
	Quercetin	Reduces MDA/lipid ROS; increases GSH	Natural product; multi-target effects	Poor bioavailability; extensive first-pass metabolism	Limited human safety data; no ICU-specific studies	(Wang et al., 2021a; Alizadeh and Ebrahimzadeh, 2022)
Novel Biologics	Anti-NINJ1 mAb	Blocks NINJ1-mediated membrane rupture	Novel mechanism; highly specific	Early preclinical stage; delivery challenges	No human data; theoretical risk: NINJ1 plays role in immune cell membrane integrity; inhibition may affect host defense	(Maremonti et al., 2024)

BBB, blood-brain barrier; GI, gastrointestinal; ROS, reactive oxygen species; GPX4, glutathione peroxidase 4; MDA, Malondialdehyde; NINJ1, ninjurin-1; xCT, cystine/glutamate antiporter; ICU, intensive care unit.

Iron chelators, including deferoxamine (DFO), deferasirox (DFX), and deferiprone, are clinically approved for the management of iron overload disorders and have recently been repurposed as inhibitors of ferroptosis. DFO has been shown to effectively reduce infarct size and improve cardiac function in myocardial ischemia–reperfusion injury (IRI) models by chelating labile iron and mitigating reactive oxygen species (ROS) generation via the Fenton reaction (Liu et al., 2022). However, DFO is limited by its short plasma half-life (20–30 minutes), poor membrane permeability, and requirement for continuous intravenous infusion (Ihnat et al., 2000; Eckes, 2011). In contrast, DFX offers oral bioavailability and a longer half-life, but is associated with renal and gastrointestinal adverse effects that may complicate management in ICU settings (Niño Taravilla et al., 2021). Deferiprone can cross the blood–brain barrier (BBB) and has shown neuroprotective effects in models of intracerebral hemorrhage, but its clinical use is limited by the risk of agranulocytosis (Cohen et al., 2000; Parakh et al., 2015).

Lipid peroxidation inhibitors, such as (Fer-1) and liproxstatin-1 (Lip-1), directly scavenge lipid radicals or inhibit lipid peroxidation chain reactions (Zilka et al., 2017). Both compounds have shown potent protective effects in diverse preclinical models, including renal IRI, traumatic brain injury, and sepsis-induced acute lung injury (Thapa et al., 2022; Xiao et al., 2026). Comparative studies suggest that Lip-1 may exhibit superior metabolic stability and potency compared to Fer-1, particularly in mitochondrial lipid peroxidation inhibition (Zilka et al., 2017). Nevertheless, both agents exhibit poor water solubility and rapid *in vivo* degradation (Zilka et al., 2017). Moreover, Fer-1 does not effectively penetrate the BBB, limiting its use in stroke or neuroinflammatory conditions (Shi et al., 2024).

Several innovative delivery systems have been developed to overcome the BBB a major obstacle for treating neurological conditions associated with ferroptosis. Shi et al. utilized RVG29, a peptide derived from rabies virus glycoprotein, to construct BBB−targeted lipid nanoparticles (T-LNPs) that encapsulate ferrostatin-1 (Fer-1) (Shi et al., 2024). These T-LNPs significantly improved BBB penetration and enhanced the neuroprotective effects of Fer-1 in a murine stroke model, as evidenced by increased fluorescence distribution in brain tissues at 6 h post−administration and reduced cerebral infarction. Wang et al. designed platelet membrane−coated liposomes (Platesome-DFO) loaded with the iron chelator deferoxamine (DFO) (Wang et al., 2025). This system leverages the natural homing ability of platelets to damaged vascular endothelium, achieving targeted delivery to the ischemic lesion, decreasing lesion iron content, and effectively inhibiting neuronal ferroptosis in cerebral ischemia/reperfusion injury models. Notably, a recent study demonstrated that liproxstatin-1 delivered via a glucose-modified macrocyclic carrier (GluAC4A) significantly improved BBB penetration and drug accumulation in the brain, reducing ferroptosis and neurological deficits in a stroke model (Geng et al., 2024). Together, these platforms are effective strategies for overcoming the BBB and enabling targeted ferroptosis inhibition in the injured brain.

Third-generation ferrostatins, such as SRS 16–86 have been developed with improved metabolic stability and improved tissue penetration (Linkermann et al., 2014). Most recently, a novel inhibitor, Ferfluor-1, was designed using a bioisosteric replacement strategy, resulting in an 8- to 40-fold increase in half-life and enabling blood-brain barrier (BBB) penetration (Yan et al., 2025). In a mouse cerebral ischemia-reperfusion injury (IRI) model, Ferfluor-1 reduced infarct volume and restored cerebral blood flow. In a Parkinson’s disease model, it successfully restored dopamine levels and improved motor function. Notably, Ferfluor-1 exhibited good safety in long-term toxicity studies, even at a high dose.

System Xc- activators and GPX4 enhancers represent another therapeutic avenue. N-acetylcysteine (NAC), a precursor of glutathione, has been widely used in critical care for indications such as acetaminophen overdose and contrast-induced nephropathy. NAC can inhibit ferroptosis by replenishing intracellular glutathione and supporting GPX4 activity, but its broad mechanism of action makes it difficult to attribute benefits specifically to ferroptosis inhibition (Zheng et al., 2025). Quercetin, a natural flavonoid, has been shown to upregulate GPX4 and SLC7A11 expression while reducing lipid ROS and iron levels in acute kidney injury models, but its poor bioavailability and extensive first-pass metabolism limit clinical translation (Wang et al., 2021a; Alizadeh and Ebrahimzadeh, 2022).

Key hurdles for clinical translation of ferroptosis inhibitors include (Kang et al., 2026; Maremonti et al., 2024): (1) Clinical trial design is impeded by the absence of appropriate biomarkers for ferroptosis detection in serum samples or tissue biopsies. (2) Most inhibitors also affect other cell death pathways, making it difficult to isolate ferroptosis-specific effects and increasing the risk of off-target toxicity. (3) Achieving therapeutic concentrations in relevant tissues, such as the brain or endothelium, without inducing systemic toxicity remains a significant hurdle. Novel delivery strategies, including nanoparticle-based carriers and hypoxia-responsive macrocyclic systems, are currently under investigation. (4) Ferroptosis plays physiological roles in immune regulation and tissue remodeling. Although ferrostatins generally exhibit a favorable side effect profile compared to ferroptosis inducers, long-term safety data in critically ill populations are still lacking. Future efforts should focus on developing more selective inhibitors, optimizing drug delivery systems, and conducting well-designed clinical trials in in critically ill populations to establish efficacy and safety.

### 5.2 Gut microbial modulators

Understanding the role of gut microbiota in ferroptosis opens up exciting possibilities for new therapeutic strategies. Interventions that restore microbial balance, such as probiotics, prebiotics, dietary modifications, and fecal microbiota transplantation (FMT), show potential for mitigating ferroptosis-associated tissue damage by reducing inflammation, enhancing antioxidant defenses, and modulating metabolite profiles. However, the efficacy and translational feasibility of these approaches vary, and key challenges must be addressed before clinical implementation in critically ill patients.

#### 5.2.1 Probiotics and prebiotics

Specific probiotic strains have shown promise in preclinical models. Administration of butyrate-producing bacteria (e.g., *Faecalibacterium prausnitzii*, *Ruminococcus*, *Roseburia*) has been shown to upregulate GPX4 and SLC7A11 in intestinal epithelial cells (Lee et al., 2025). These beneficial commensals are typically depleted in ICU patients, with studies demonstrating reduced abundances of SCFA-producers such as *Faecalibacterium*, *Blautia*, and *Bifidobacterium* during critical illness (Lee et al., 2025). *Lactobacillus* and *Bifidobacterium* strains have been reported to enhance antioxidant capacity and reduce inflammation in animal models of sepsis (Takeaki, 2025).

However, clinical studies evaluating microbiome-targeted interventions in critically ill patients have yielded inconsistent results. The largest randomized controlled trial to date, which assessed probiotics for ventilator-associated pneumonia, failed to show clinical benefit. A comprehensive meta-analysis with trial sequential analysis also concluded that further studies on pro/synbiotics in critically ill patients are unlikely to demonstrate any significant benefit (Lee et al., 2023). Additionally, safety concerns are notable, which reports of extra-intestinal seeding following live microbial therapeutics have dampened enthusiasm for these interventions, particularly in immunocompromised ICU patients (Williams, 2010).

#### 5.2.2 Dietary interventions and novel microbial products

Omega-3 polyunsaturated fatty acids (n-3 PUFAs) and fiber-rich diets can modulate gut microbiota composition and metabolite production. Butyrate and n-3 PUFAs enhance the expression of antioxidant enzymes including GPX4 and thioredoxin reductase 1(TXNRD1), protecting cells from iron-dependent cell death (Saschin et al., 2026). However, dietary interventions in critically ill patients are complicated by altered gastrointestinal function and feeding intolerance (Bachmann et al., 2025).

A promising novel approach is the use of pasteurized (inactivated) microbial products. The PAM-ICU trial (NL-OMON57972, https://onderzoekmetmensen.nl/en/trial/57972) is currently investigating whether daily oral supplementation with pasteurized Akkermansia muciniphila (PAM) in ICU survivors with recent sepsis is safe and can increase the abundance of beneficial anaerobic bacteria. This inanimate microbial product has been shown to reinforce gut barrier integrity, reduce inflammation, and is classified as safe by EFSA. If effective, this approach may support intestinal recovery and reduce long-term complications in ICU survivors.

#### 5.2.3 Fecal microbiota transplantation

FMT from healthy donors has been shown to restore microbial diversity in various conditions (Shimizu et al., 2025). However, its application in critically ill patients faces significant challenges, including a lack of standardized protocols, risk of transmitting multidrug-resistant organisms, and potential for inducing systemic inflammatory responses (Karimianghadim et al., 2025).

In addition, A phased approach to microbiome−targeted therapy in ICU patients is proposed. First: Limit further dysbiosis and prevent pathogen expansion by avoiding unnecessary antibiotics, providing enteral nutrition (fiber−rich if tolerated), and early SCFA support (Ghosh et al., 2025; Serbanescu et al., 2025). Second: Restore microbial diversity and enhance barrier function using probiotics if not severely immunocompromised, prebiotics, and butyrate supplementation. Third: Achieve full microbial reconstitution and prevent long-term complications by considering fecal microbiota transplantation (FMT) for refractory dysbiosis and continued dietary support (Mullish et al., 2024). Special consideration: Antibiotic stewardship throughout the ICU stay minimizes microbiota disruption by using de-escalation protocols and, whenever possible, pathogen-specific rather than broad-spectrum antibiotics. Future precision-based approaches should stratify patients by: (1) dysbiosis severity (alpha diversity, pathogen abundance), (2) immune status (neutrophil count, lymphocyte subsets), (3) antibiotic exposure history, and (4) gut barrier function. For severely immunocompromised patients (e.g., neutropenia, recent transplant), live biotherapeutics are generally contraindicated; pasteurized microbial products (e.g., *Akkermansia muciniphila* in the PAM-ICU trial) represent a safer alternative.

Key challenges for clinical translation include the lack of consistent benefit, as large RCTs and systematic meta-analyses have failed to demonstrate efficacy of prebiotics/probiotics in ICU patients despite encouraging signals in individual studies (Lee et al., 2023). Safety concerns are particularly pronounced in immunocompromised hosts, where disrupted intestinal barriers and impaired immunity increase the risk of microbial translocation and infection with live biotherapeutics (Williams, 2010; Shimizu et al., 2025). Furthermore, current “one-size-fits-all” approaches lack specificity, as they are poorly targeted to the heterogeneous types of dysbiosis observed across different ICU patients and phases of care (Lee et al., 2025). Antibiotic interference further complicates treatment, as broad-spectrum antibiotics, which are ubiquitous in ICU settings, profoundly disrupt gut microbiota and may limit the engraftment and efficacy of administered probiotics or FMT (Shimizu et al., 2025). Individual variability in baseline microbiota composition, underlying disease, and concurrent medications also influences treatment response (Alverdy et al., 2024). Finally, mechanistic uncertainty persists, as the causal relationship between specific microbial metabolites and ferroptosis regulation in humans remains to be established (Gao et al., 2025).

## 6 Limitations and future perspectives

Despite growing recognition of ferroptosis in vascular injury, several fundamental limitations hinder its clinical translation.

First, validated biomarkers for ferroptosis are critically lacking, which represents a major impediment to clinical trial design. Commonly used experimental markers in preclinical studies include lipid peroxidation products (malondialdehyde [MDA], 4-hydroxynonenal [4-HNE]), iron indices (Fe²^+^/Fe³^+^ ratio, ferritin), glutathione system parameters (GSH/GSSG ratio, GPX4 activity, SLC7A11 expression), ferroptosis regulatory proteins (GPX4, ACSL4, PTGS2, xCT), genetic markers (SNPs in GPX4/SLC7A11), lipidomics signatures (oxidized phospholipids). However, translating these markers to ICU settings faces multiple technical challenges (Michel et al., 2008; Chen et al., 2021a): (1) Specificity: MDA and 4-HNE are elevated in any condition involving oxidative stress (e.g., sepsis, trauma), not exclusively ferroptosis; (2) Stability: Lipid peroxidation products are highly reactive with short half-lives, requiring immediate sample processing-difficult in routine ICU workflows; (3) Tissue availability: GPX4 expression assays require tissue biopsies, rarely feasible in critically ill patients; (4) Standardization: No standardized cut-off values or reference ranges exist across hospitals; (5) Real-time monitoring: Most assays cannot be adapted for bedside or rapid-turnaround clinical laboratory use. The development of robust, clinically applicable biomarkers (ideally detectable in biofluids like plasma or urine) that can accurately quantify ferroptosis activity, predict therapeutic response, and monitor target engagement remains the most urgent priority for translating ferroptosis-targeted therapies into the ICU (He et al., 2026).

Second, the gut microbiota-ferroptosis axis in humans remains poorly defined. While preclinical studies link microbial metabolites to ferroptosis regulators (SCFAs modulating GPX4, TMAO influencing ACSL4), these findings await validation in human pathophysiology. Causal relationships and context-dependent effects in critically ill patients require systematic clinical investigation. We emphasize that direct experimental evidence supporting causality in humans is currently lacking and that the proposed “axis” should be regarded as a working hypothesis requiring rigorous prospective validation.

Third, a significant translational gap persists between preclinical efficacy and clinical application. Key hurdles include achieving optimal drug delivery to target tissues without inducing systemic toxicity, ensuring tissue specificity, and establishing long-term safety. Future research must prioritize the development of innovative delivery systems, such as nanoparticle-based carriers or hypoxia-responsive macrocyclic carriers, which hold promise for overcoming biological barriers like the blood-brain barrier and enabling targeted therapy. Furthermore, the inherent heterogeneity of critically ill populations, with variations in disease etiology, stage, and concurrent medications (e.g., broad-spectrum antibiotics), poses a substantial challenge for trial design, necessitating biomarker-stratified patient selection and adaptive trial protocols.

Fourth, in the complex pathophysiology of the ICU setting, particularly in sepsis, multiple regulated cell death (RCD) pathways including apoptosis, pyroptosis, necroptosis, and ferroptosis, contribute to tissue injury and organ failure, but they do not operate in isolation. Emerging evidence indicates extensive crosstalk and hierarchical interactions among these pathways (Wu et al., 2025; Zou et al., 2026) (**Table 2**). Apoptosis, primarily driven by caspase family activation, is the dominant leukocyte death program associated with late-phase immune depletion and immunosuppression in sepsis. In contrast, pyroptosis (mediated by inflammasomes and gasdermin D) and necroptosis (mediated by RIPK1/RIPK3/MLKL) amplify early hyperinflammatory responses by inducing membrane permeabilization and release of damage-associated molecular patterns (DAMPs). Ferroptosis, characterized by iron-dependent lipid peroxidation, operates through distinct mechanisms but its downstream execution involves NINJ1-mediated membrane rupture-a terminal pathway shared with pyroptosis and necroptosis (Bao et al., 2026).

**Table 2:** Crosstalk between ferroptosis and other regulated cell death pathways (Bao et al., 2026; Wu et al., 2025; Zou et al., 2026).

Cell death pathway	Key mediators	Primary role in sepsis	Interaction with ferroptosis
Apoptosis	Caspase-3/8, Bcl-2 family	Late-phase immunosuppression; leukocyte depletion	Ferroptosis evasion in apoptotic-resistant cells; caspase-8 inhibits RIPK1, limiting ferroptosis-necroptosis crosstalk
Pyroptosis	Caspase-1/11, GSDMD, NLRP3	Early hyperinflammation; DAMP release (IL-1β, IL-18)	Ferroptosis-mediated lipid peroxidation promotes NLRP3 activation; both converge on NINJ1-mediated membrane rupture
Necroptosis	RIPK1, RIPK3, MLKL	Inflammatory cell lysis; organ injury amplification	Shared NINJ1-mediated membrane rupture; iron accumulation sensitizes to TNF-induced necroptosis
Ferroptosis	GPX4, ACSL4, iron, lipid ROS	Endothelial barrier disruption; vascular leakage	NINJ1 executes membrane rupture in ferroptosis; can be blocked by NINJ1 inhibition

ACSL4, acyl-CoA synthetase long-chain family member 4; Bcl-2, B-cell lymphoma 2; Caspase-1/11, cysteine-aspartic protease 1/11; Caspase-3/8, cysteine-aspartic protease 3/8; DAMP, damage-associated molecular pattern; GPX4, glutathione peroxidase 4; GSDMD, gasdermin D; IL-1β, interleukin-1 beta; IL-18, interleukin-18; MLKL, mixed lineage kinase domain-like protein; NLRP3, NOD-like receptor family pyrin domain containing 3; NINJ1, nerve injury-induced protein 1; RIPK1, receptor-interacting serine/threonine-protein kinase 1; RIPK3, receptor-interacting serine/threonine-protein kinase 3; ROS, reactive oxygen species; TNF, tumor necrosis factor.

In summary, translating ferroptosis-targeted therapies to clinical practice requires concerted efforts in biomarker development, mechanistic human studies, innovative delivery strategies, and a deeper understanding of cell death crosstalk. Addressing these limitations through interdisciplinary collaboration will determine whether the therapeutic potential of ferroptosis modulation can be realized in critically ill patients.

## 7 Conclusion

Ferroptosis has emerged as a critical pathogenic mechanism in vascular injury among critically ill patients, driving endothelial dysfunction and organ failure in conditions such as ischemia-reperfusion injury, stroke, atherosclerosis, and sepsis. The identification of a “gut-ferroptosis-vascular axis”, whereby gut microbiota-derived metabolites regulate key pathways including GPX4, SLC7A11, and ACSL4, opens new therapeutic avenues. While targeting ferroptosis through inhibitors or microbiota-based interventions holds significant promise, translating these insights into clinical practice requires addressing critical gaps, including the development of specific biomarkers, validation in humans, and rigorous evaluation in well-designed trials. A deeper understanding of this axis could establish a new paradigm for managing vascular injury, with interdisciplinary efforts bridging mechanistic insights and clinical translation potentially reducing mortality and enhancing recovery in critically ill patients.
